# Negative thermal expansion induced by intermetallic charge transfer

**DOI:** 10.1088/1468-6996/16/3/034904

**Published:** 2015-06-02

**Authors:** Masaki Azuma, Kengo Oka, Koichiro Nabetani

**Affiliations:** 1 Materials and Structures Laboratory, Tokyo Institute of Technology, 4259 Nagatsuta, Midori-ku, Yokohama, 226-8503, Japan; 2 Department of Applied Chemistry, Faculty of Science and Engineering, Chuo University, 1-13-27 Kasuga, Bunkyo-ku, Tokyo 112-8551, Japan

**Keywords:** negative thermal expansion, perovskite, charge transfer, x-ray diffraction

## Abstract

Suppression of thermal expansion is of great importance for industry. Negative thermal expansion (NTE) materials which shrink on heating and expand on cooling are therefore attracting keen attention. Here we provide a brief overview of NTE induced by intermetallic charge transfer in A-site ordered double perovskites SaCu_3_Fe_4_O_12_ and LaCu_3_Fe_4−*x*_Mn_*x*_O_12_, as well as in Bi or Ni substituted BiNiO_3_. The last compound shows a colossal dilatometric linear thermal expansion coefficient exceeding −70 × 10^−6^ K^−1^ near room temperature, in the temperature range which can be controlled by substitution.

## 1. Introduction

**Figure 1.**
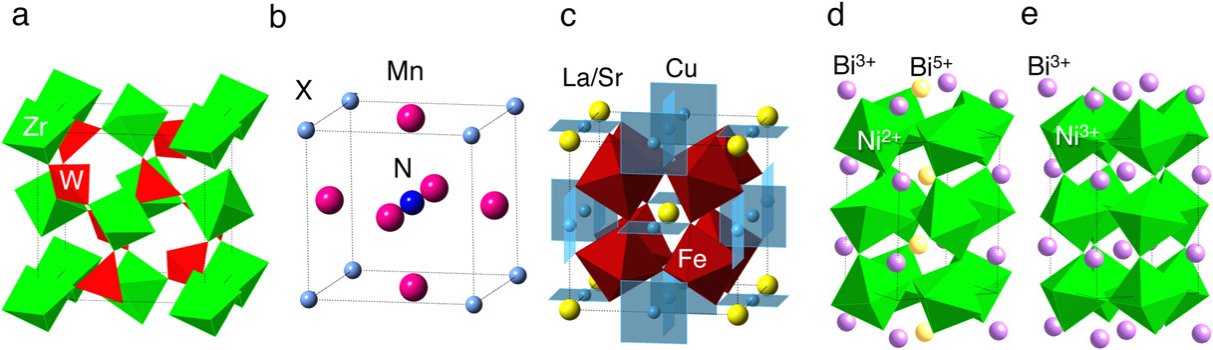
Crystal structures of NTE compounds. ZrW_2_O_8_ (a), anti-perovskite manganese nitride Mn_3_XN (b), ACu_3_Fe_4_O_12_ (c) and ambient-pressure and high-pressure phases of BiNiO_3_ (d) and (e).

Thermal expansion originating from anharmonic vibration of atoms is a common feature of matter in solid, liquid and gas states. For example, the coefficient of linear thermal expansion (CTE) of iron is *α*_L_ = (1/*L*)(*ΔL*/*ΔT*) = −11.6 × 10^−6^ K^−1^ leading to the 1.16 *μ*m expansion of a 10 cm long rod on heating by 1 K. Nanoscale production of electronic devices and optical communications requires precise positioning, and thus, even such small amounts of thermal expansion can be a problem. Negative thermal expansion (NTE) materials which shrink on heating and expand on cooling are attracting much interest because these are expected to compensate for the thermal expansion of structure materials by making composites [1–4]. Useful NTE materials for zero or controlled expansion composites should show a smooth contraction while heating through a wide temperature range. The compounds with flexible frameworks in the crystal structures (mechanism 1) such as *β*-LiAlSiO_4_ [1, 2] ZrW_2_O_8_ [5], and Cd(CN)_2_ [6] can be categorized into the first generation of NTE materials. Indeed, crystallized glass, where *β*-LiAlSiO_4_ crystallizes into a Li–Al–Si–O glass matrix, is widely used as a low thermal expansion material in cooktops and astronomical telescopes. The last decade has seen a remarkable development in materials with NTE resulting from phase transitions. In particular, a large NTE over *α*_L_ = −30 × 10^−6^ K^−1^ coupled with a magnetic transition (mechanism 2) was discovered in an anti-perovskite manganese nitride [7–13]. PbTiO_3_ based perovskites were found to show NTE originating from a ferroelectric-paraelectric transition (mechanism 3) [14–19]. An intermetallic charge transfer transition was shown to cause volume shrinkage (mechanism 4) in A-site ordered double perovskites upon heating [20–23]. Colossal dilatometric linear thermal expansion coefficient over −70 × 10^−6^ K^−1^ is observed in the controlled temperature range near room temperature (RT) in Bi or Ni substituted perovskite compound BiNiO_3_ [24–26]. The properties of these typical NTE materials are summarized in table 1, and the crystal structures of selected compounds are shown in figure 1. In this review, we focus on the NTE induced by intermetallic charge transition in A-site ordered double perovskites and modified BiNiO_3_.

**Table 1.** Coefficients of linear thermal expansion and operating temperatures *T*_oper_ of typical negative thermal expansion materials.

Materials	*α* (×10^−6^ K^−1^)	*T*_oper_ (K)	Mechanism	Method^a^	References
*β*-LiAlSiO_4_	−1 to −6	300–900	1	D	[1, 2]
ZrW_2_O_8_	−6	425–1030	1	D/N	[5]
Cd(CN)_2_	−33.5	170–375	1	D	[6]
Mn_3_Cu_0.53_Ge_0.47_N	−16	265–340	2	D	[9]
Mn_3.1_Zn_0.5_Sn_0.4_N	−36.4	335–375	2	D	[13]
PbTiO_3_	−6.6	298–367	3	X	[14]
0.5PbTiO_3_–0.5BiFeO_3_	−13.5	300–600	3	X	[19]
SrCu_3_Fe_4_O_12_	−22.6	220–230	4	X	[21]
Bi_0.95_La_0.05_NiO_3_	−82	320–380	4	D	[24]
Bi_0.95_Nd_0.05_NiO_3_	−134	380–410	4	D	[25]

a D: dilatometry, N: neutron diffraction, X: x-ray diffraction.

## 2. NTE induced by intermetallic charge transfer in A-site ordered double perovskites

**Figures 2.**
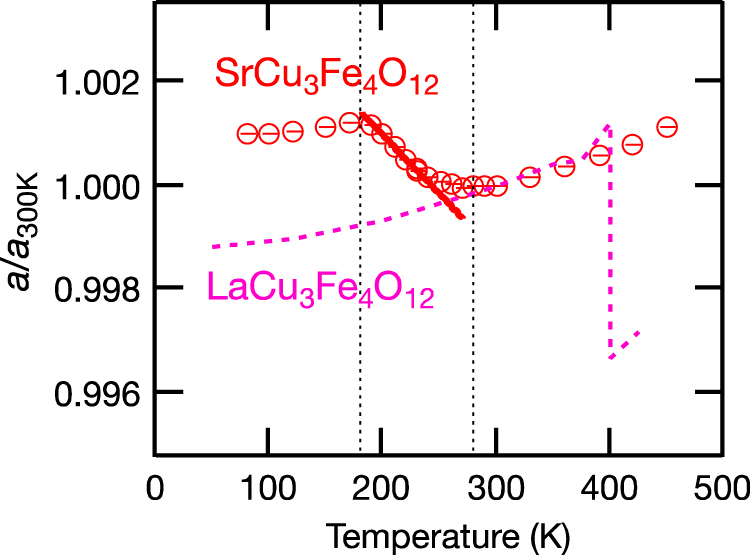
Temperature dependence of lattice constants of LaCu_3_Fe_4_O_12_ and SrCu_3_Fe_4_O_12_ determined by XRD measurements. Reproduced with permission from [21], copyright 2011 John Wiley and Sons.

**Figure 3.**
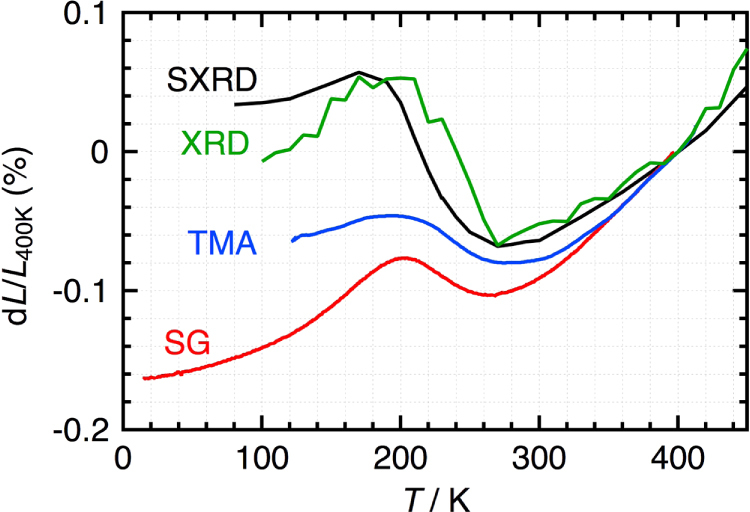
Thermal expansion of SrCu_3_Fe_4_O_12_ evaluated by XRD, SXRD, TMA and strain gauge. Reproduced with permission from [22].

**Figure 4.**
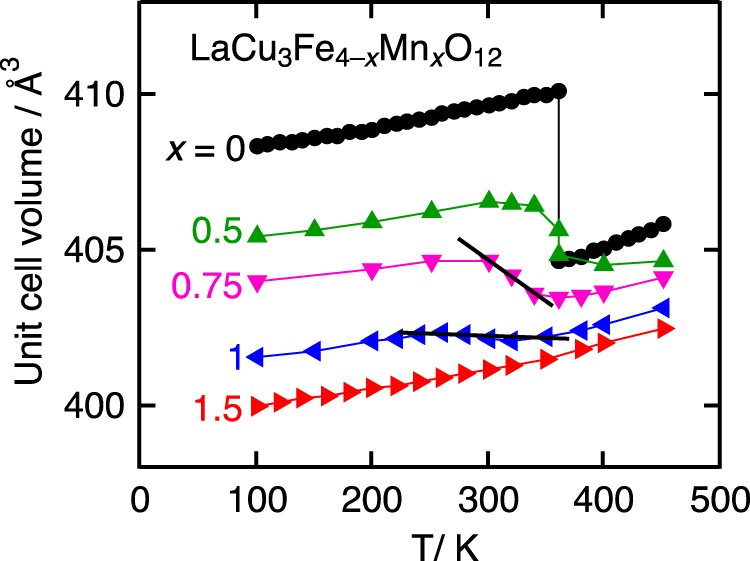
Temperature dependence of unit cell volumes of LaCu_3_Fe_4−*x*_Mn_*x*_O_12_ determined by XRD measurements. Reprinted with permission from [23], copyright 2014, AIP Publishing LLC.

LaCu_3_Fe_4_O_12_ has so-called A-site ordered double perovskite structure where A-site of perovskite ABO_3_ is periodically occupied by La^3+^ and Cu^3+^ as shown in figure 1(c). The formal valence state of this compound is La^3+^Cu^3+^_3_Fe^3+^_4_O_12_ at RT. Intermetallic charge transfer between Cu^3+^ and Fe^3+^ takes place on heating above 393 K resulting in La^3+^Cu^2+^_3_Fe^3.75+^_4_O_12_ high-temperature (HT) phase. Because of the shrinkage of Fe–O bonds, the unit cell volume shrinks by 1% [20]. This transition is discontinuous first order one as shown in figure 2 and the thermal expansion coefficient cannot be defined. However, replacement of La^3+^ by Sr^2+^ changes the nature of transition to second order and NTE with *α*_L_ = 22.6 × 10^−6^ K^−1^ is observed between 200 and 230 K as shown in figure 2 [21]. The NTE was confirmed by dilatometric measurements with thermal mechanical analysis (TMA) and strain gauge (figure 3) [22]. Similar NTE is also realized in LaCu_3_Fe_4−*x*_Mn_*x*_O_12_ as shown in figure 4 [23].

## 3. NTE in A- or B- site substituted perovskite compound BiNiO_3_

### 3.1. Pressure induced intermetallic charge transfer in BiNiO_3_

BiNiO_3_ is a perovskite compound with a triclinically distorted crystal structure (space group P-1) stabilized by high-pressure (HP) synthesis at 6 GPa. Bi is a main group element, but it has Bi^3+^/Bi^5+^ charge degree of freedom depending on 6s^2^ and 6s^0^ electronic configurations. These occupy distinct crystallographic sites in BiNiO_3_ whose valence distribution is unusual Bi^3+^_0.5_Bi^5+^_0.5_Ni^2+^O_3_ as illustrated in figure 1(d) [27]. Powder neutron diffraction (PND) [28] and x-ray absorption spectroscopy (XAS) [29] studies revealed a pressure-induced melting of the Bi-charge disproportionation at 3–4 GPa and a simultaneous Ni to Bi charge transfer accompanied by a structural change to the orthorhombic GdFeO_3_ type perovskite superstructure (figure 1(e)) with valence distribution Bi^3+^Ni^3+^O_3_ and an insulator to metal transition. The structural transition is accompanied by a discrete shrinkage of lattice parameters and a 2.5% decrease in the unit cell volume [24]. This large change results from the dominant contraction of the Ni–O perovskite framework as Ni^2+^ is oxidized to the smaller Ni^3+^ at the transition, which outweighs the lattice expanding effects of reducing Bi^5+^ to Bi^3+^ and increases in the Ni–O–Ni angles.

**Figure 5.**
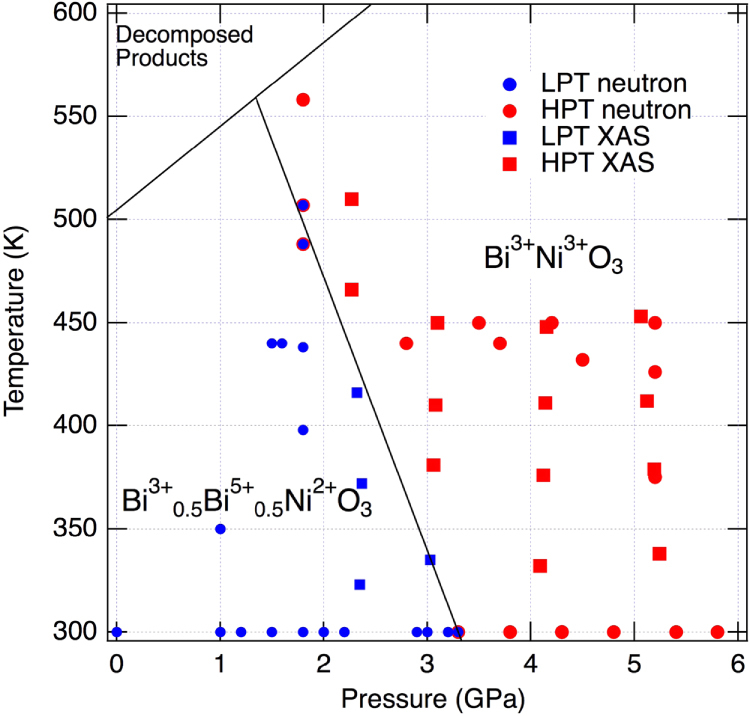
Pressure–temperature phase diagram of BiNiO_3_ determined by PND and XAS studies. Circles and squares show PND and XAS data, and blue and red symbols correspond to the low pressure and temperature (LPT) and high pressure and temperature (HPT) phases, respectively. Reproduced from [24].

The PND and XAS results have been used to construct the *P*–*T* phase diagram for BiNiO_3_ shown in figure 5. BiNiO_3_ decomposes above 500 K at ambient pressure (AP), but is stabilized up to at least 565 K at 1.8 GPa (and to ∼1300 K at 6 GPa under synthesis conditions). The boundary between the low pressure and temperature (LPT) and HPT phases has slope d*T*_CT_/d*p* = –140 KGPa^−1^. The 2.5–3.4% volume contraction occurs on both pressurizing and heating.

### 3.2. NTE in Bi_0.95_La_0.05_NiO_3_

The large *ΔT*_CT_ of BiNiO_3_ shows that colossal NTE is feasible but the transition is only observed above pressures of 1.5 GPa in pure BiNiO_3_. Chemical substitutions for Bi is used to suppress the charge disproportionation in the Bi^3+^_0.5_Bi^5+^_0.5_Ni^2+^O_3_ phase and thereby shift the charge transfer transition to near ambient conditions. Partial substitution of La^3+^ without charge degree of freedom for Bi destabilizes the characteristic Bi^3+^/Bi^5+^ disproportionation and shift the charge transfer transition to around 350 K at AP in Bi_0.95_La_0.05_NiO_3_ [24, 30].

**Figure 6.**
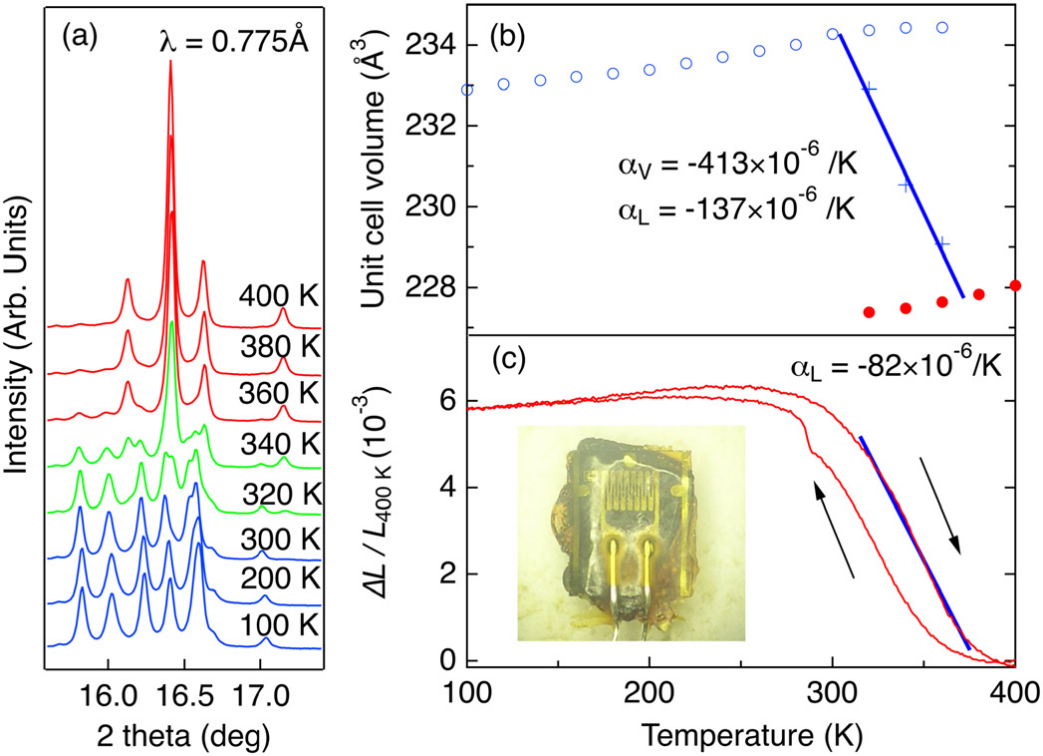
Selected SXRD data of Bi_0.95_La_0.05_NiO_3_ at various temperatures (a). Reproduced from [30]. Temperature dependence of the unit cell volume (b). The dilatometric linear thermal expansion of Bi_0.95_La_0.05_NiO_3_ on heating and cooling (c) The inset shows the sample pasted on the strain gauge. Reproduced from [24].

Synchrotron x-ray diffraction (SXRD) patterns on Bi_0.95_La_0.05_NiO_3_ in figure 6(a) show merging of five main peaks characteristic for the triclinic phase with Bi^3+^/Bi^5+^ charge disproportionation into three indicating the transition to the orthorhombic phase with (Bi, La)^3+^Ni^3+^O_3_ valence distribution. The 2.9% volume shrinkage, which has a similar magnitude to that observed in undoped BiNiO_3_ under a pressure of 1.8 GPa, was observed as shown in figure 6(b). Coexistence of the low and high temperature phases is observed at three points in the transition region and a linear fit to the weighted average volumes is used to obtain the transition width of *ΔT*_CT_ = 70 K. Such a coexistence of two phases changing the fractions as functions of temperature appears to against the Gibbs phase rule, but is commonly observed in ZrO_2_ and HfO_2_ [31]. In these ceramics, low-temperature monoclinic and HT tetragonal phases coexist changing the phase fractions across the diffusionless (martensitic) phase transition. The deviation of the pressure from 1 atm at the domain boundary is thought to be the origin of such phenomena. The crystallographic volume thermal expansion coefficient of Bi_0.95_La_0.05_NiO_3_ between 300 and 370 K is *α*_V_ = – 413 × 10^−6^ K^−1^ and the linear coefficient is 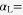
–137 × 10^−6^ K^−1^, showing that colossal NTE magnitudes are observable in Bi_1−*x*_La_*x*_NiO_3_. Crystallography predicts the upper limit of the magnitude of thermal expansion as the formation of pores and other microstructural defects can lessen the effect in bulk ceramics.

Dilatometric measurements on a polycrystalline ceramic of Bi_0.95_La_0.05_NiO_3_ prepared at HP were made during heating and cooling cycles as shown in figure 6(c). The strain *ΔL*/*L*(400 K) increases with increasing temperature up to 270 K indicating the normal positive thermal expansion, but decreases above 270 K. The average observed *α*_L_ between 270 and 400 K is −49 × 10^−6^ K^−1^ and the maximum negative slope between 320 and 380 K corresponds to a linear thermal expansion coefficient of −82 × 10^−6^ K^−1^ [24]. It was confirmed that the oxidation of Ni ion from 2+ to 3+ was the origin of this volume shrinkage by XAS measurement [32].

### 3.3. Tunable NTE in Bi_1−*x*_Ln_*x*_NiO_3_ (Ln: Lanthanides)

**Figure 7.**
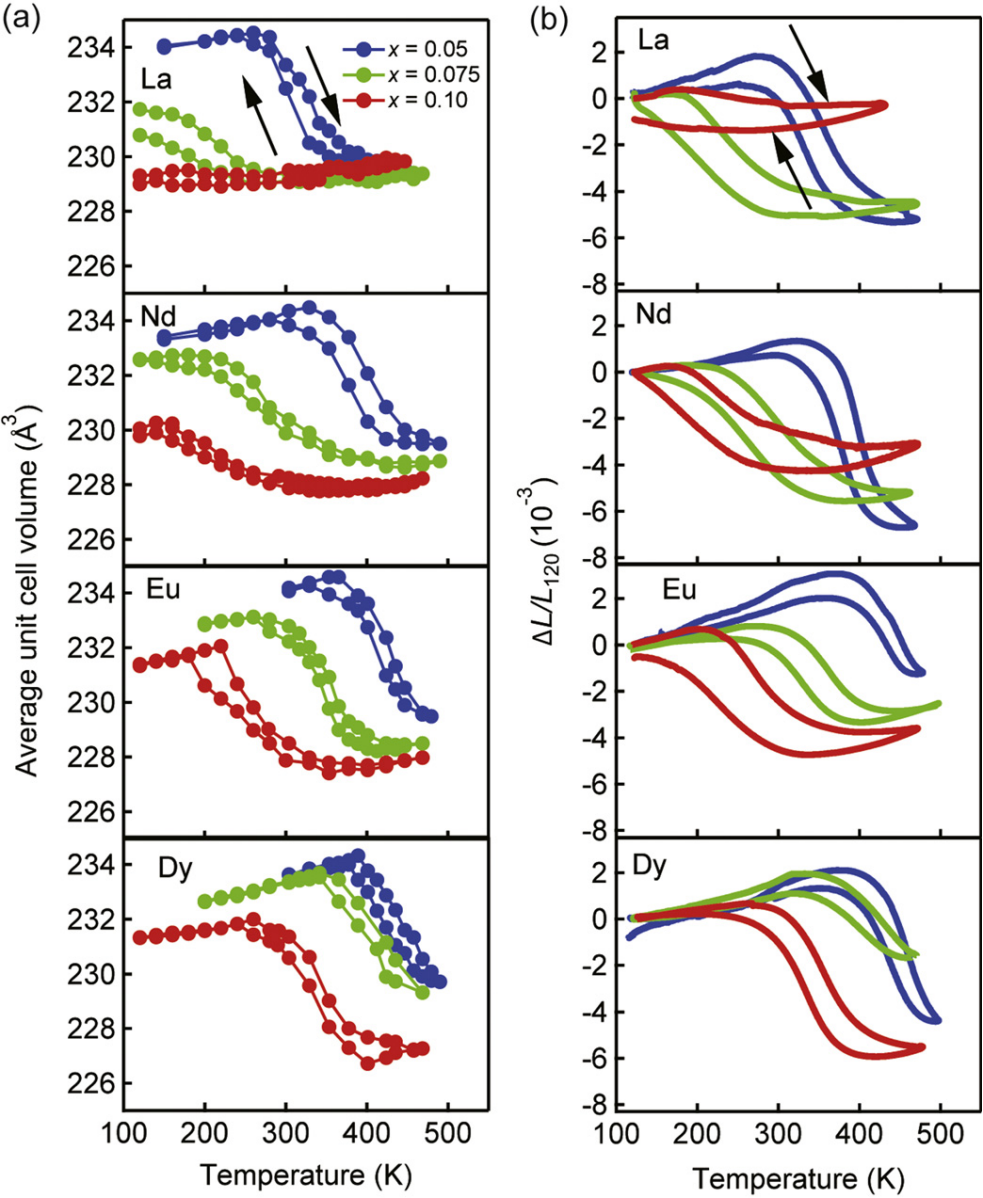
Temperature dependence of the weighted average volume (a) and the dilatometric linear thermal expansion (b) of Bi_1-*x*_Ln_*x*_NiO_3_ (Ln = La, Nd, Eu, Dy; *x* = 0.05, 0.075, 0.10) on heating and cooling. Reproduced from [25].

**Figure 8.**
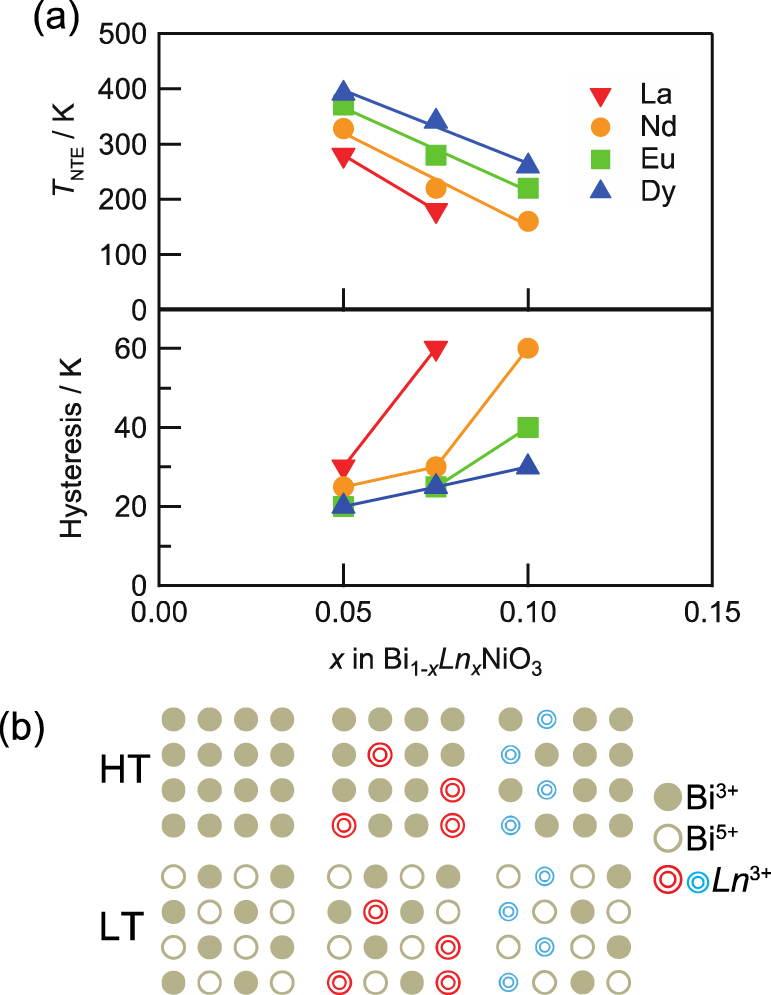
(a) Composition dependence of *T*_NTE_ on heating and temperature hysteresis width determined by XRD. (b) Illustration of the Bi/Ln layer of BiNiO_3_ (left) and Bi_1−*x*_Ln_*x*_NiO_3_ (center and right) in the HT orthorhombic phase and low temperature triclinic phase (LT). Filled and open circles represent valence of 3**+** and 5**+**, respectively. The large Ln^3**+**^ ions (red) are distributed homogeneously. On the other hand, the small Ln^3**+**^ ions (blue) have partial ordering. Reproduced from [25].

The temperature range of NTE and the CTE can be controlled by tuning the composition, i.e., the element substituting Bi and the degree of substitution. Thermal expansion of Bi_1−*x*_Ln_*x*_NiO_3_ (Ln: = La, Nd, Eu, Dy) were investigated [25]. Figure 7(a) shows the temperature dependence of the weighted average volume calculated from the unit cell volumes and the phase fractions refined by Rietveld analysis of laboratory XRD data. NTE with temperature hysteresis is present in all samples. The dilatometric curves measured by TMA (figure 7(b)) are consistent with the volume change. Table 2 summarizes the NTE parameters determined from the TMA data. For all systems, the temperature range of NTE upon heating shifts to the lower side and the volume shrinkage becomes more gradual as the Ln^3+^ content increases. Figure 8(a) summarizes the compositional dependence of the onset temperature of NTE (*T*_NTE_) and the temperature hysteresis width. The *T*_NTE_ decreases almost linearly with the amount of substituted lanthanide, and this result shows that the temperature range can be controlled by chemical tuning. The transition temperature also depends on the ionic radius of Ln^3**+**^. Substitution with a small Ln^3**+**^ stabilizes the triclinic phase and maintains it at a HT. This lanthanide dependence is explained as follows. Under HPT synthesis conditions, Bi_1−*x*_Ln_*x*_NiO_3_ is in the orthorhombic (Bi, Ln)^3+^Ni^3+^O_3_ state with an unique Bi/Ln site. The La^3**+**^ ions are homogeneously distributed, because the ionic radius of La^3**+**^ is close to that of Bi^3**+**^. Suppose such a sample is in the HT (Bi, Ln)^3**+**^Ni^3**+**^O_3_ state at AP. Charge disproportionation on cooling is suppressed by the presence of a La^3**+**^ ion at the Bi^5**+**^ site of the triclinic low-temperature phase. *T*_NTE_ is therefore lowered by the La substitution, and the orthorhombic phase is dominant for Bi_0.90_La_0.10_NiO_3_. On the other hand, a large difference in ionic radius between Bi^3**+**^ and small Ln^3**+**^ ions should lead to a partial Bi^3+^/Ln^3**+**^ ordering, as schematically illustrated in figure 8(b). The charge transfer transition is less affected by a small Ln^3+^ because Bi^5+^ can periodically exist owing to the Ln/Bi ordering. Since the amount of substitution is small, the partial ordering is not observed as a super structure. La substitution decreases not only the transition temperature, but also the sharpness of the transition, which is also a consequence of randomness. The unit cell volume and *ΔL*/*L* curves for Bi_1−*x*_Dy_*x*_NiO_3_ in figure 7 show parallel shifts to a lower temperature with increasing *x*. Most importantly, the temperature hysteresis summarized in figure 8(a) is reduced by using smaller lanthanides.

**Table 2.** The liner thermal expansion coefficient *α*_L_ of Bi_1−*x*_Ln_*x*_NiO_3_ (Ln = La, Eu, Nd, Dy) estimated by the dilatometric measurement on heating [25].

Ln	*x* in Bi_1−*x*_Ln_*x*_NiO_3_	*T* (K)	*α*_L_ (10^−6^ K^–1^)
La	0.05	330 → 380	−71
	0.075	220 → 280	−36
	0.10	210 → 300	−5
Nd	0.05	380 → 410	−134
	0.075	280 → 330	−41
	0.10	200 → 250	−30
Eu	0.05	430 → 460	−70
	0.075	330 → 390	−41
	0.10	250 → 310	−44
Dy	0.05	440 → 470	−104
	0.075	410 → 460	−43
	0.10	330 → 380	−68

### 3.4. NTE in LaNi_1−*x*_M_*x*_O_3_ (M: Al and Ga)

**Figure 9.**
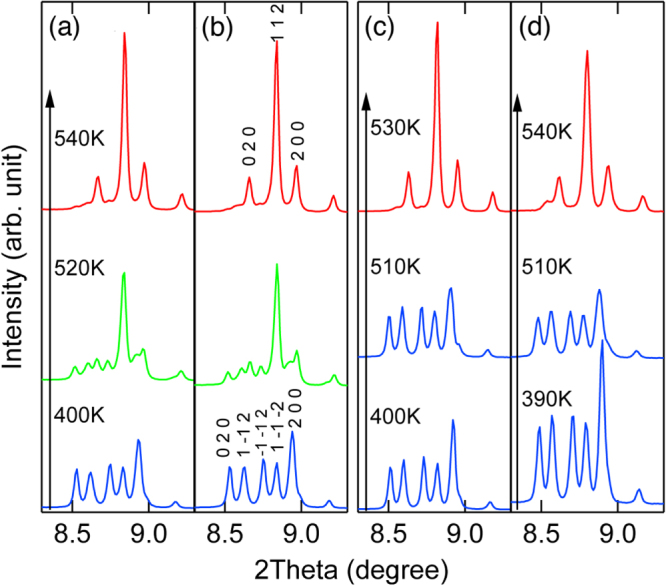
SXRD patterns of BiNi_0.925_Al_0.025_O_3_ (a), BiNi_0.9_Al_0.1_O_3_ (b), BiNi_0.95_Ga_0.05_O_3_ (c) and BiNi_0.9_Ga_0.01_O_3_ (d) at various temperatures revealing the transition from triclinic to orthorhombic phases on heating.

The above discussed NTE results from the temperature induced intermetallic charge transfer between Bi^5+^ and Ni^2+^. The presence of Ln^3+^ in Bi^5+^ site destabilizes the Bi^3+^/Bi^5+^ charge disproportionation in Bi^3+^_0.5_Bi^5+^_0.5_Ni^2+^O_3_ and HP phase of Bi^3+^Ni^3+^O_3_ appears on heating at AP. In this context, substitution of Ni^2+^ with a trivalent ion is also expected to stabilize Bi^3+^(Ni, M)^3+^O_3_ and thus leads to NTE. Figure 9 shows the XRD patterns of BiNi_1−*x*_M_*x*_O_3_ (M = Ga, Al) on heating. They reveal transitions from triclinic to orthorhombic phases via coexistence of two phases, essentially the same as those for Bi_1−*x*_Ln_*x*_NiO_3_.

**Figure 10.**
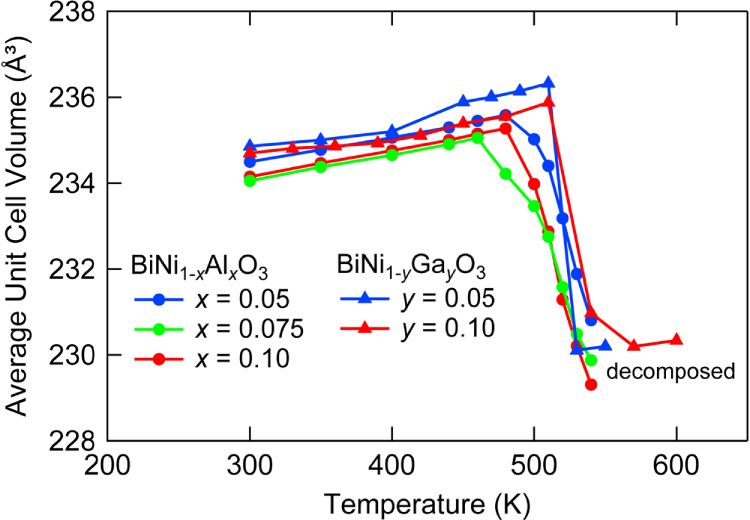
Temperature dependence of the weighted average volume of BiNi_1−*x*_Al_*x*_O_3_ (*x* = 0.05, 0.075, 0.10) and BiNi_1-*x*_Ga_*x*_O_3_ (*x* = 0.05, 0.10) measured on heating.

The weighted average unit cell volumes obtained by the Rietveld analysis of the XRD data are plotted in figure 10. Note that only the data on heating are plotted since the samples partially decomposed after heating up to 550 K. These results indicate the presence of NTE, but *T*_NTE_ is at around 500 K, well above RT and are almost independent of *x*. It is recently found that BiNi_1-*x*_Fe_*x*_O_3_ also shows large NTE with *α*_L_ exceeding −150 × 10^−6^ K^−1^ in the controlled temperature range near RT [26].

## 4. Conclusions

A brief overview of NTE induced by intermetallic charge transfer in A-site ordered double perovskites SrCu_3_Fe_4_O_12_ and LaCu_3_Fe_4−*x*_Mn_*x*_O_12_ and A- or B-site substituted perovskite BiNiO_3_ is provided. The distinct volume contraction in LaCu_3_Fe_4_O_12_ is broadened by replacement of La^3+^ by Sr^2+^ or Mn substitution for Fe, leading to NTE. Substitution of Bi with Ln^3+^ or Ni with Al^3+^, Ga^3+^ stabilized the (Bi, Ln)^3+^(Ni, M)^3+^O_3_ phase which is present only in the HP condition for pure BiNiO_3_ and suppress the intermetallic charge transfer transition accompanied by volume contraction to ambient condition. Colossal NTE with CTE over −70 × 10^−6^ K^−1^ is observed by both diffraction and dilatometric measurements in the controlled temperature range for Bi_1−*x*_Ln_*x*_NiO_3_ while *T*_NTE_ is almost independent for BiNi_1−*x*_Al_*x*_O_3_ and BiNi_1−*x*_Ga_*x*_O_3_. These compounds are promising for the suppression of the thermal expansion of structure materials, but the presence of emperature hysteresis owing to the first-order toransition is a problem for the practical applications. It is recently shown that the thermal hysteresis is suppressed in BiNi_1−*x*_Fe_*x*_O_3_. Moreover, 18 vol. % addition of BiNi_0.85_Fe_0.15_O_3_ with *α*_L_ = −187 × 10^−6^ K^−1^ compensates for the thermal expansion of epoxy resin [26].
